# Assessment of Economic Burden of Concurrent Measles and Rubella Outbreaks, Romania, 2011–2012

**DOI:** 10.3201/eid2506.180339

**Published:** 2019-06

**Authors:** Joseph Njau, Denisa Janta, Aurora Stanescu, Sarah S. Pallas, Adriana Pistol, Nino Khetsuriani, Susan Reef, Daniel Ciurea, Cassandra Butu, Aaron S. Wallace, Laura Zimmerman

**Affiliations:** Centers for Disease Control and Prevention, Atlanta, Georgia, USA (J. Njau, S.L. Pallas, S. Reef, A.S. Wallace, L. Zimmerman);; National Public Health Institute, Bucharest, Romania (D. Janta, A. Stanescu, A. Pistol);; Centers for Disease Control and Prevention, Tbilisi, Georgia (N. Khetsuriani);; Center for Health Policies and Services, Bucharest (D. Ciurea);; World Health Organization Country Office, Bucharest (C. Butu)

**Keywords:** measles, rubella, measles virus, rubella virus, viruses, congenital rubella syndrome, economic burden, concurrent outbreaks, outbreak costs, middle-income countries, Romania

## Abstract

We estimated the economic impact of concurrent measles and rubella outbreaks in Romania during 2011–2012. We collected costs from surveys of 428 case-patients and caretakers, government records, and health staff interviews. We then estimated financial and opportunity costs. During the study period, 12,427 measles cases and 24,627 rubella cases were recorded; 27 infants had congenital rubella syndrome (CRS). The cost of the outbreaks was US $9.9 million. Cost per case was US $439 for measles, US $132 for rubella, and US $44,051 for CRS. Up to 36% of households needed to borrow money to pay for illness treatment. Approximately 17% of patients continued to work while ill to pay their treatment expenses. Our key study findings were that households incurred a high economic burden compared with their incomes, the health sector bore most costs, and CRS costs were substantial and relevant to include in rubella outbreak cost studies.

Outbreaks of measles and rubella constitute serious public health events and entail a vigorous response from public health agencies. A typical response could include a range of complex activities, including isolation of case-patients, case–contact tracing, assessment of disease or vaccination history of each case-patient, identification of potentially susceptible persons, and, if required, vaccination or quarantine (*1*). Economic impact studies of measles and rubella outbreaks in high-income countries illustrate a high cost of outbreaks and response activities (*1*–*5*). However, little information is available on the economic impact of measles and rubella outbreaks in a middle-income country, and in 2011, this impact was identified as a global research priority (*6*). Concurrent measles and rubella outbreaks in Romania provided an opportunity to estimate this economic impact.

In coordination with other World Health Organization European Region countries, Romania has a goal of measles and rubella elimination by 2020. During 2000–2003, coverage with a first dose of measles-containing vaccine in Romania was reported to be >95%; for 2004–2010, combined measles and rubella vaccine first dose coverage was >95% and second dose coverage was >88%. In spite of these high national coverage rates among recent birth cohorts, unvaccinated subpopulations exist among older age cohorts and subnationally, which create conditions for continued measles or rubella outbreaks.

During 2011–2012, Romania experienced concurrent outbreaks of measles and rubella. Measles cases were primarily reported among children from the northwest part of the country, and rubella cases were reported primarily among adolescents and adults throughout Romania. The outbreaks resulted in 12,427 measles cases and 24,627 rubella cases during 2011–2012 (the number of measles cases officially associated with the outbreak was subsequently revised to 12,234 after our analysis was conducted) (*7*). We estimated the economic cost of these outbreaks and response activities incurred by the health sector and households of persons with measles or rubella infection.

## Methods

### Definitions and Costs

We assessed the cost of the measles and rubella outbreaks by collecting data on direct and indirect costs from households and the health sector. We defined economic cost as the sum of financial costs (i.e., the monetary value of goods and services provided to treat case-patients and to contain the outbreak) and opportunity costs (i.e., the value of the best alternative forgone by the health sector or households caused by measles or rubella illness or treatment) by using the societal costing perspective. The health sector included government entities from primary to tertiary level (i.e., health facilities, agencies, or departments) involved in providing treatment to measles or rubella case-patients and in designing and implementing the public health response to the outbreaks.

### Data Sources and Collection Process

#### Household Direct and Indirect Costs

To collect household costs, we conducted a survey among a purposely selected sample of households that had recent cases (within the previous 18 months to minimize recall bias) drawn from the national measles and rubella surveillance database (Table 1; Appendix Figure). An initial sample of 1,217 persons met the inclusion criteria; of these, 789 case-patients or their representatives could not be reached or declined to participate and 428 were interviewed (response rate 35%).

**Table 1:** Definitions and data sources used for cost components during assessment of economic burden of concurrent measles and rubella outbreaks, Romania, 2011–2012*

Cost component	Definition	Data sources
Healthcare provider	All reimbursements for diagnostic testing for measles and rubella, inpatient and outpatient care, emergency treatment, case management	Reimbursement data from National Health Insurance House (CNAS) covering all registered government healthcare providers using the Romanian national clinical diagnosis codes associated with measles and rubella diagnoses, outpatient and inpatient care, and vaccinations (Appendix Table), as submitted by healthcare providers
National outbreak response	Enhanced surveillance, laboratory diagnosis and confirmation, field activities such as case investigations, and outbreak response immunization, including actual expenses incurred for personnel, laboratory supplies, and vaccine doses provided to contacts of cases and to high-risk communities	Interviews with key personnel, including the Director of the National Institute for Public Health, Romanian MOH; the Team Leads for Measles and Rubella, National Center for Surveillance and Control of Communicable Diseases, MOH; a measles and rubella epidemiologist from the District Public Health Authorities, MOH; the Director of the reimbursement department and an analyst at the National Health Insurance House (CNAS); and the Head of Country Office and a public health officer at the World Health Organization country office in Romania
Direct medical and nonmedical household	Medical costs (e.g., consultation fees, laboratory tests and medications) and nonmedical costs (e.g., transport, food and lodging) paid out-of-pocket by cases and caregivers	Household survey of 428 recent case-patients (within last 18 months) by using structured telephone interviews
Indirect household	Workdays lost by cases and caregivers because of measles or rubella infections	Household survey of 428 recent case-patients (within last 18 months) by using structured telephone interviews; 2012 Romanian national minimum wage of 700 Lei/month was used as proxy to quantify the value of lost workdays by patients and caretakers >18 y of age (*8*).
CRS cases healthcare provider	All diagnostic services, inpatient and outpatient care reimbursements related to CRS diagnostic codes for identified CRS cases during timeframe	Reimbursement data from National Health Insurance House (CNAS) covering all registered government healthcare providers for the 27 CRS cases identified from the time of the outbreak up to the date of data collection; reimbursement data were obtained for 18 (11 surviving and 7 who had died) of the 27 case-patients
CRS cases lifetime discounted indirect	Workdays lost by CRS cases over assumed lifetime productive working years (18–63 y)	2012 Romanian national minimum wage of 700 Lei/month was used as a proxy to quantify the value of lost workdays by patients >18 y of age (*8*)

*CNAS, Casa Nationala de Asigurari de Sanatate; CRS, congenital rubella syndrome; MOH, Ministry of Health.

#### Healthcare Provider Costs

Reimbursement data were obtained from the National Health Insurance House (Casa Nationala de Asigurari de Sanatate), which covered all registered government healthcare providers in Romania (Table 1). There were ≈10,000 primary care physicians or family doctors and >500 secondary or tertiary hospitals in the 42 health districts in Romania (*8*). Reimbursement fees are typically paid when doctors and hospitals submit claims to national health insurance funds for the service provided.

#### National Outbreak Response Costs

To assess national outbreak response costs, personnel involved in outbreak response activities were interviewed on the basis of referral from the National Institute for Public Health, Romanian Ministry of Health, which was charged with coordinating the national outbreak response. These open-ended interviews were conducted by members of the research team in Romanian at the offices of respondents and focused on collecting information about actual expenses incurred in measles and rubella outbreak response activities, including costs of enhanced surveillance, outbreak investigation, and outbreak response vaccination and vaccine stockpiling (Table 1).

#### Congenital Rubella Syndrome Costs

We requested reimbursement data for health services received by the 27 identified congenital rubella syndrome (CRS) cases from birth up to the date of data collection (at which point the surviving 15 CRS case-patients were 3–4 years old) from the National Health Insurance House; we obtained records for 18 (11 surviving and 7 who had died) of the 27 case-patients. The projection of their lifetime productivity losses used the same data sources and definitions as the main analysis of costs during the outbreak (Table 1). These 27 CRS case-patients were the only CRS cases included in the calculation of CRS costs during the outbreak. 

All data collection activities occurred from mid-January through the end of May 2014. A post-hoc analysis of the costs of CRS cases was conducted in March–May 2016.

### Data Analysis

We analyzed data by using Access and Excel (Microsoft, https://www.microsoft.com), and Stata version 13 (StataCorp LLC, https://www.stata.com). We calculated district-level weights on the basis of the number of measles and rubella cases as a proportion of the total population in each of the 42 districts of Romania. We then applied these weights to the household survey data, with the costs reported by a respondent multiplied by the weight of the district in which that respondent resided, to estimate the mean, median, and interquartile range of treatment expenditures per household for measles and rubella infections and the proportion of cases receiving treatment by admission status.

We calculated the indirect costs of measles or rubella infections by first calculating the number of measles and rubella case-patients and caregivers >18 years of age who were employed (based on employment rates reported in the patient survey). We then assumed that each of these employed case-patients and caregivers >18 years of age lost the average number of workdays missed on the basis of the household survey. We valued each day of work missed at the minimum wage of Romania.

We used a human capital approach to estimate the lifetime productivity losses for CRS case-patients (*9*). We assumed that had these children not been born with CRS, their working years would have been 18–63 years of age (until median retirement age in Romania) with the same labor force participation, employment rates, minimum wage, and exchange rate during these years as those used in the main analysis (i.e., constant 2012–2013 rates). We discounted lifetime productivity losses to 2013 US dollars by using a 3% discount rate. 

All costs were adjusted for inflation by using the Romanian Consumer Price Index to 2013 prices and converted from Romanian local currency (Lei) into US dollars by using 2013 exchange rates (3.32 Lei/1 US dollar). Costs are presented in 2013 US dollars (*10*,*11*).

### Sensitivity Analysis

We conducted univariate sensitivity analysis to characterize how total costs would change with different input values to reflect uncertainty. Reported number of outbreak cases, healthcare provider costs, and national outbreak response costs varied by ± 10%. Out-of-pocket treatment costs, number of days of work missed by cases and caregivers, and CRS case direct medical costs varied from the 25th to 75th percentiles, and proportion of cases receiving care varied from the 5th to 95th percentiles of the sample distribution. CRS case indirect costs were also calculated from birth to an average life expectancy of 74 years.

### Ethics Approval and Informed Consent

The assessment protocol was approved by the Romanian National Health Research Ethics Review Board and determined not to be human subjects research by the US Centers for Disease Control and Prevention. Informed consent was obtained from participants after the objectives of the assessment were described to them. Participants were informed that the information would be confidential, there were no unique identifiers, and that participation was voluntary. The authors certify that this study has been conducted in an ethical way according to the international standards for authors.

## Results

A retrospective review of the Romanian national measles and rubella surveillance database showed that 12,427 laboratory-confirmed and epidemiologically linked cases of measles and 24,627 laboratory-confirmed and epidemiologically linked cases of rubella were reported during the 2011–2012 outbreaks (Figure). In addition, 27 confirmed cases of CRS were reported.

**Figure:**
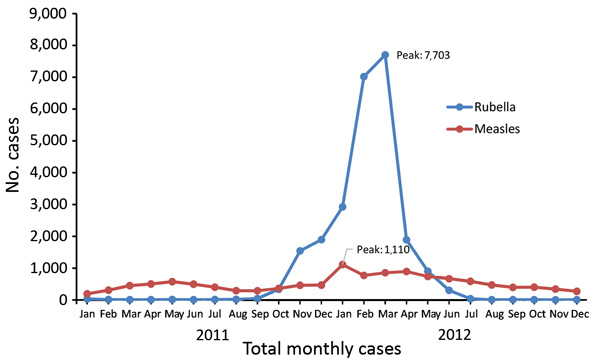
Reported cases of measles and rubella during concurrent outbreaks, Romania, 2011–2012. Values reflect revision of official case counts after analysis was completed.

### Household Survey Results

The number of households with measles or rubella cases that were included in the survey was evenly split between urban and rural areas, and 46% of case-patients were employed (Table 2). Although approximately one third of responding measles or rubella case-patients were <18 years of age, more case-patients in this age range were infants and preschoolers for measles (85%) than for rubella (44%). A slightly higher proportion of rubella case-patients (10%) sought care on multiple occasions compared with measles case-patients (4%). However, measles case-patients were 3.5 times more likely to have been admitted to a hospital than rubella case-patients (Table 2). Few measles or rubella case-patients (<2%) sought care at a private health facility. The distribution of sampled case-patients by sex was similar to those for the outbreaks overall, whereas in terms of age the sampled case-patients included a higher share of adults >18 years of age than the outbreaks overall.

**Table 2:** Descriptive characteristics of case-patients responding to household survey for assessment of economic burden of concurrent measles and rubella outbreaks, Romania, 2011–2012

Characteristic	No. (%) case-patients		
	Measles	Rubella	Total
Total sample	219	209	428
Age <18 y	74 (34)	63 (30)	137 (32)
Male sex	109 (50)	107 (51)	216 (50)
Urban domicile	98 (55)	115 (45)	213 (50)
Multiple care visits	8 (4)	21 (10)	29 (7)
Admission to hospital	185 (84)	38 18)	223 (52)
Care at private clinics	3 (1)	4 (2)	7 (2)
Age >18 y	145	146	291
Formal or informal employment	72 (50)	63 (43)	135 (46)
Students	13 (9)	66 (45)	79 (27)
Retired, unemployed or housewives	60 (41)	17 (12)	77 (26)
Age <18 y	74	63	137
Infants and preschoolers	63 (85)	28 (44)	91 (66)
School children	11 (15)	35 (56)	46 (34)
Treatment expenses	219	209	428
Incurred some treatment expenses	206 (94)	194 (93)	400 (93)
Borrowed money for treatment expenses	79 (36)	14 (7)	93 (22)

#### Household Direct Medical and Nonmedical Costs

More than 90% of responding households with either measles or rubella cases incurred some type of treatment expense (Table 2). Case-patients with measles (36%) and rubella (7%) reported having to borrow money to pay for costs related to an episode of measles or rubella (Table 2). The largest share of household spending was on medications, followed by transportation. We found no notable differences in transportation or overall spending for households residing in rural areas compared with those in urban settings, which might reflect the history of widespread geographic availability of healthcare by state-owned polyclinics throughout Romania. Average household spending for measles and rubella patients <18 years old was more than that for patients >18 years old (US $112.51 vs. US $45.85). Weighted median inpatient costs for measles were 2.2 times higher than for rubella, and outpatient costs for measles were 1.5 times higher than for rubella (Table 3). Total estimated direct medical and nonmedical household costs for treatment were US $888,338 for the 12,427 measles cases and US $477,261 for the 24,627 rubella cases (Table 4).

**Table 3:** Main analysis input and sensitivity analysis values used for cost analysis of economic burden of concurrent measles and rubella outbreaks, Romania, 2011–2012*

Cost component	Disease	Input	Input value	Median (25th–75th percentiles)	Proportion (95% CI)	Sensitivity analysis LB	Sensitivity analysis UB
Case distribution	Measles	No. cases	12,427	NA	NA	11,184	13,670
	Rubella	No. cases	24,627	NA	NA	22,164	27,090
	Rubella	No. CRS cases	27	NA	NA	NA	NA
	Rubella	Patients who died as of May 2016	12	NA	NA	NA	NA
	Rubella	Patients who survived as of May 2016	15	NA	NA	NA	NA
	Measles	Cases in 2011, %	39	NA	NA	NA	NA
	Measles	Cases in 2012, %	61	NA	NA	NA	NA
	Rubella	Cases in 2011, %	16	NA	NA	NA	NA
	Rubella	Cases in 2012, %	85	NA	NA	NA	NA
Healthcare provider	Measles	Reimbursement to healthcare providers for diagnostic codes, 2011	$1,313,049	NA	NA	$1,181,744	$1,444,353
	Measles	Reimbursement to healthcare providers for diagnostic codes, 2012	$1,962,708	NA	NA	$1,766,437	$2,158,979
	Rubella	Reimbursement to healthcare providers for diagnostic codes, 2011	$107,494	NA	NA	$96,745	$118,244
	Rubella	Reimbursement to healthcare providers for diagnostic codes, 2012	$567,138	NA	NA	$510,425	$623,852
National outbreak response	Measles	Personnel time for outbreak response	$5,164	NA	NA	$4,648	$5,680
	Measles	Reagents/tests for outbreak response	$449,225	NA	NA	$404,303	$494,148
	Measles	Emergency vaccine purchases	$61,962	NA	NA	$55,766	$68,158
	Rubella	Personnel time for outbreak response	$10,437	NA	NA	$9,393	$11,481
	Rubella	Reagents/tests for outbreak response	$907,951	NA	NA	$817,156	$998,746
	Rubella	Emergency vaccine purchases	$125,235	NA	NA	$112,712	$137,759
Direct medical and nonmedical household	Measles	Out-of-pocket cost for inpatient care	NA	$66.87 ($21.27–$110.94)	NA	$21.27	$110.94
	Rubella	Out-of-pocket cost for inpatient care	NA	$30.70 ($15.20–$82.06)	NA	$15.20	$82.06
	Measles	Out-of-pocket cost for outpatient care	NA	$18.23 ($4.55–$39.51)	NA	$4.55	$39.51
	Rubella	Out-of-pocket cost for outpatient care	NA	$12.50 ($6.07–$24.32)	NA	$6.07	$24.32
	Measles	Patients receiving outpatient care, %	NA	NA	19 (24–60)	24	60
	Measles	Patients receiving inpatient care, %	NA	NA	81 (80–109)	80	100
	Rubella	Patients receiving outpatient care, %	NA	NA	62 (17–27)	17	27
	Rubella	Patients receiving inpatient care, %	NA	NA	38 (24–91)	34	91
Indirect household	Measles	Employment rate for case-patients and caregivers, %	34	NA	NA	NA	NA
	Rubella	Employment rate for case-patients and caregivers, %	32	NA	NA	NA	NA
	Measles	No. work days missed by case-patients	NA	11.45 (7–18)	NA	7	18
	Rubella	No. work days missed by case-patients	NA	9.62 (7–14)	NA	7	14
	Measles	Case caregivers who missed work, %	89	NA	NA	NA	NA
	Rubella	Case caregivers who missed work, %	68	NA	NA	NA	NA
	Measles	No. work days missed by caregivers	NA	7 (3–10)	NA	3	10
	Rubella	No. work days missed by caregivers	NA	4.5 (3–7)	NA	3	7
	Both	Minimum daily wage	$10.44	NA	NA	NA	NA
CRS, direct medical	Rubella	Reimbursement to healthcare providers for patients who survived	NA	$6,455 ($1,749–$10,296)	NA	$1,749	$10,296
	Rubella	Reimbursement to healthcare providers for patients who died	NA	$2,777 ($1,734–$3,309)	NA	$1,734	$3,309
CRS, lifetime discounted indirect	Rubella	Minimum daily wage	$10.44	NA	NA	NA	NA
	Rubella	Years (range) of productivity loss	46 (18–63)	NA	NA	NA	74 (LE)
	Rubella	Labor force participation rate, %	65	NA	NA	NA	100
	Rubella	Unemployment rate, %	11	NA	NA	NA	0
	Rubella	Discount rate, %	3	NA	NA	NA	NA

*Monetary values are in US 2013 dollars. CRS is an outcome of rubella. CRS, congenital rubella syndrome; LB, lower bound; LE, life expectancy; NA, not applicable; UB, upper bound.

**Table 4:** Estimated total costs by component for base case and sensitivity analyses for assessment of economic burden of concurrent measles and rubella outbreaks, Romania, 2011–2012*

Cost component	Disease or outcome	Main analysis base case	Sensitivity analysis variable	Sensitivity analysis LB	Sensitivity analysis UB	Total measles and rubella costs with LB	Total measles and rubella costs with UB
Healthcare provider	Measles	$3,275,757	Healthcare provider costs	$2,948,181	$3,603,332	$9,555,968	$10,211,120
	Rubella	$674,633	Healthcare provider costs	$607,169	$742,096	$9,816,081	$9,951,007
National outbreak response	Measles	$516,351	National outbreak response costs	$464,716	$567,986	$9,831,909	$9,935,179
	Rubella	$1,043,623	National outbreak response costs	$939,261	$1,147,985	$9,779,182	$9,987,906
Direct medical and nonmedical household	Measles	$883,338	Out-of-pocket inpatient and outpatient treatment costs	$266,573	$1,572,347	$9,266,779	$10,572,553
			Proportion of patients receiving inpatient or outpatient treatment	$716,509	$915,498	$9,716,715	$9,915,704
			No. cases	$795,004	$971,672	$9,795,210	$9,971,878
	Rubella	$477,261	Out-of-pocket inpatient and outpatient treatment costs	$141,497	$1,136,431	$9,547,779	$10,542,713
			Proportion of patients receiving inpatient or outpatient treatment	$310,302	$768,764	$9,716,584	$10,175,046
			No. cases	$429,535	$524,988	$9,835,818	$9,931,270
Indirect household	Measles	$779,917	No. work days missed by case-patients	$583,614	$1,068,856	$9,687,241	$10,172,484
			No. work days missed by caregivers	$622,875	$897,698	$9,726,502	$10,001,325
	Rubella	$1,043,281	No. work days missed by case-patients	$827,714	$1,403,658	$9,667,976	$10,243,920
			No. work days missed by caregivers	$959,358	$1,183,153	$9,799,621	$10,023,416
CRS cases direct medical	Rubella	$130,143	Reimbursement to healthcare providers for CRS cases	$47,038	$194,145	$9,800,438	$9,947,546
CRS cases lifetime discounted indirect	Rubella	$1,059,241	Productive years (labor force participation, unemployment rate)	NA	$2,165,257	NA	$10,989,560
Total	Measles	$5,455,363	NA	NA	NA	NA	NA
	Rubella	$4,428,182	NA	NA	NA	NA	NA
	Both	$9,883,545	NA	NA	NA	NA	NA

*CRS, congenital rubella syndrome; LB, lower bound; NA, not applicable; UB, upper bound.

#### Household Indirect Costs

Among responding patients participating in the labor market, a median of 11.45 days were lost for measles and 9.62 days for rubella (Table 3). The maximum number of workdays lost were 68 days for a single episode of measles and 21 days for an episode of rubella. Of those >18 years of age, 27% reported working while ill. Among those who continued to work, the average number of days worked while ill with measles or rubella was 4 days. Students and schoolchildren reported an average of 10 days that they were unable to attend school because of measles or rubella infection. Total estimated indirect household costs were US $779,917 for the 12,427 measles cases and US $1,043,281 for the 24,627 rubella cases (Table 4).

#### Total Estimated Household Costs

We determined total estimated direct and indirect household costs. These values were US $1.7 million for 12,427 measles cases (US $133.84/case) and US $1.5 million for 24,627 rubella cases (US $61.74/case) (Tables 4, 5).

**Table 5:** Estimated overall cost per case of measles, rubella, or congenital rubella syndrome during concurrent outbreaks, Romania, 2011–2012*

Cost type	Cost per case (sensitivity analysis lower bound–upper bound; 2013 US $)		
Measles	Rubella, not CRS	CRS
Household direct medical and nonmedical	$71 ($21–$127)	$19 ($6–$46)	NA
Household indirect	$63 ($47–$86)	$42 ($34–$57)	NA
Healthcare provider	$264 ($237–290)	$27 ($25–30)	NA
National outbreak response	$42 ($37–46)	$42 ($38–47)	NA
CRS cases direct medical	NA	NA	$4,820 ($1,742–$7,191)
CRS cases lifetime discounted indirect	NA	NA	$39,231 (UB: $80,195)
Estimated total societal/case	$439 ($389–494)	$132 ($118–$158)	$44,051 ($40,973–$85,015)

*CRS, congenital rubella syndrome; NA, not applicable; UB, upper bound of sensitivity analysis.

### Healthcare Provider Costs

Healthcare providers received direct reimbursement fees from health insurance. These totals were US $3,275,757 for services provided to treat measles and US $674,633 for services provided to treat rubella infections during the outbreak (Table 4).

### National Outbreak Response Costs

National response activities included enhanced surveillance, laboratory diagnostic testing, and immunization of at-risk populations at a total cost of US $1,559,975, of which ≈60% were for activities related to diagnosis and containment of rubella (Table 4). Virtually equal amounts of national resources were spent on measles and rubella containment efforts per case (US $41.55/case for measles and US $42.38/case for rubella) (Table 5). Most (87%) costs were incurred for laboratory reagents, laboratory tests, and overtime salaries for laboratory technicians (Table 3).

### CRS Case Costs

Estimated health service reimbursement costs for the 27 CRS cases up to May 2016 were US $130,143 (Table 4). Estimated discounted lifetime productivity losses for the 12 CRS case-patients who had died by May 2016 were US $470,774. Under the assumption that the 15 surviving CRS case-patients would also be unable to work during their lifetimes, their estimated discounted lifetime productivity losses would be US $588,467 (US $1,059,241 for all 27 case-patients) (Table 4).

### Societal Costs

Total societal costs of the measles and rubella outbreaks were US $9.9 million, of which US $5.5 million was for measles-related activities and US $4.4 million for rubella-related activities (the rubella-related activities included US $1.2 million in CRS-related costs) (Table 4). These costs translated to a total cost per case of US $439 for measles, US $132 for rubella (not including CRS), and US $44,051 for CRS (Table 5). Sensitivity analysis showed that total costs varied between US $9,266,779 (when the lower bound of measles treatment costs was used) and US $10,989,560 (when the upper bound of lifetime CRS case indirect costs was used) (Table 4).

## Discussion

The large concurrent outbreaks of measles and rubella in Romania provided a major opportunity for an economic assessment of both diseases, as well as for CRS, in a middle-income country setting. Our key study findings were that households incurred a high economic burden of measles or rubella infection compared with income, the health sector bore most of the economic cost of the measles and rubella outbreaks, and CRS case costs were substantial and relevant to include in rubella outbreak cost studies. Our study contributes to the limited literature on measles and rubella outbreak costs in middle-income countries and to the evidence gap in this global research priority.

The economic burden of the measles and rubella outbreaks on the outbreak-affected households was substantial. Almost every household incurred costs relating to measles or rubella treatment. The direct household cost per case of measles or rubella was high compared with the average income in Romania in 2012: 30% of monthly income and 3% of annual income for measles, 9% of monthly income and 1% of annual income for rubella. Previous studies indicated that, when illness-related economic costs are >10% of annual household income, it is considered catastrophic because it potentially forces households to cut consumption of necessities, such as food and water, and leads to increased debt or greater poverty (*12*–*14*). Although direct household costs per case in our study in general did not exceed this threshold in Romania (≈US $263 in 2013), the highest cost households did; moreover, these costs were for only 1 medical event, and the financial burden would increase if other illnesses were considered.

For households, the unexpected measles and rubella treatment costs had to be met in the short term. High medical costs incurred in a short period are considered more detrimental than high costs incurred over a long period (*12*). Approximately 36% of households with measles and 7% with rubella reported borrowing money to pay for these expenses. Similar medical bill coping strategies, including informal payments, have also been confirmed in other studies in Romania (*15*,*16*). As a consequence of these expenses, studies in Romania and other countries in eastern Europe have reported that up to 60% of patients are forced to postpone or completely forgo treatments because of lack of money to pay their medical bills (*15*,*17*). Because per capita health spending in Romania was less than that in the United States (US $580) during 2013 (*18*), the estimated direct household and health sector costs per case of measles (US $376) and rubella (US $89, not including CRS costs) were high.

The health sector bore 70% of the economic cost of the outbreak for measles and 39% for the outbreak of rubella. For measles, 86% of the health sector costs were for provider reimbursement. For rubella, 61% was for national outbreak response activities. More than 85% of the national outbreak response activities were for laboratory reagents and test kits for measles and rubella, but only 12% was for emergency vaccine purchase because large-scale supplemental immunization activities were not conducted. Assuming a cost per dose administered of US $5, the cost of vaccinating all 36,861 confirmed case-patients with measles or rubella with 2 doses of the measles and rubella vaccine before the outbreak would have cost the health sector 10% of the amount paid for health sector costs for the measles outbreak and 21% of the amount paid for health sector costs for the rubella outbreak. Any preoutbreak vaccination campaign would have had to vaccinate more than just the 36,861 persons who were infected during the outbreak to prevent the outbreak, and the costs of strategies to reach persistently unvaccinated subpopulations in Romania might be higher than typical campaign or routine immunization costs. Even so, such preemptive campaigns to reduce immunity gaps, coupled with continued robust routine immunization delivery, would have provided protection and reduced costs to the health sector, not only during the 2011–2012 outbreaks but also for future outbreaks. This finding is critical because Romania continues to have measles outbreaks.

Our study contributes to the ongoing efforts to document evidence of the economic burden of vaccine-preventable diseases in middle-income countries, with particular focus on measles and rubella. A study from Ethiopia reported an estimated household cost of US $29.18/case of measles treated during a measles outbreak in 2011 (*2*). As in Romania, the Ethiopian health sector bore most (80%) of the outbreak cost. A study in Latin America found the costs of measles treatment to range from US $43 in Nicaragua to US $210 in Argentina; the average cost was US $190 for the entire Latin America region (*19*). As an upper-middle-income country, economic costs for Romania are more comparable to those reported for countries such as the Netherlands, the United Kingdom, and Canada; a study from these countries in 2002 reported the societal costs of measles treatment as US $254 in Canada, US $276 in the Netherlands, and US $307 in the United Kingdom (*20*).

Our post-hoc analysis of CRS direct medical costs and estimated productivity losses adds to the limited literature on CRS costs in different economic settings (*21*). Studies from Brazil, Oman, and Uzbekistan reported that estimated CRS costs ranged from US $18,644 to >US $1.18 million/case (*22*–*24*). More than 90% of these costs constitute indirect CRS costs. Future studies of CRS economic burden in low- and middle-income countries are needed. In this assessment, we did not quantify the value of life lost because of measles or rubella infections occurring during the outbreak, aside from the CRS cost estimates.

Our cost estimates are subject to several limitations. The household cost estimates were retrospective, and some persons might have been susceptible to recall bias. The purposive sampling of recent cases and district-level weighting procedures used for the household survey do not necessarily provide a nationally representative estimate of household costs, and no adjustment was made to account for survey nonresponse (response rate 35%), which might introduce selection bias; household costs might be underestimated because of the smaller proportion of case-patients <18 years of age in our sample compared with the outbreaks overall. We only recorded the provider reimbursement claims that were actually paid by the national insurance companies. However, anecdotal information suggested that claims were sometimes reimbursed at lower standard amounts based on limited availability of funds at the national level. National outbreak response costs were based on interviews with key informants from the agencies responsible for response implementation and are believed to be exhaustive of the costs incurred. However, other agencies might have also provided outbreak response and might not have been known to the national health authorities we used as key informants for the agencies involved in outbreak response.

For the CRS cost analysis, we did not obtain information on 9 of the reported cases (response rate 67%), which might introduce selection bias in our results, although no systematic differences in clinical profiles between included and excluded cases were observed. Information on some types of future CRS care costs (e.g., special schooling, government disability payments) were not available and are therefore not included in the analysis. The CRS productivity losses assume that all surviving case-patients are fully unable to work; this assumption might overestimate the indirect costs of CRS if some of these case-patients are ultimately able to work for income. Because of the nonrepresentative sampling techniques used and nonresponse, no uncertainty bounds were calculated for these cost estimates. Finally, the estimation of economic costs was not fully comprehensive. The opportunity costs of healthcare workers devoting their time to doing other public health activities was not estimated because of incomplete data.

In conclusion, the costs of the 2011–2012 measles and rubella outbreaks in Romania were high, especially when compared with total average household incomes and national health expenditures per capita. In addition to the health consequences of these outbreaks, households faced major threats of financial insecurities during these outbreaks and the long-term economic impacts of productivity losses. Preventing outbreaks through routine vaccination also reduces the economic burden on the health sector. This study makes a major contribution to identifying the overall societal economic burden of measles and rubella outbreaks in Romania and improves our understanding of the magnitude of the costs of measles and rubella outbreaks in middle-income economies.

Appendix Additional information on assessment of economic burden of concurrent measles and rubella outbreaks, Romania, 2011–2012.
