# Supplementary material for: Assessment of Economic Burden of Concurrent Measles and Rubella Outbreaks, Romania, 2011–2012
Source: Emerg Infect Dis. 2019 Jun;25(6):1101–9. doi: 10.3201/eid2506.180339 (PMC6537719; doi:10.3201/eid2506.180339)
Supplement: Appendix — Additional information on assessment of economic burden of concurrent measles and rubella outbreaks, Romania, 2011–2012. [file 18-0339-Techapp-s1.pdf]

Article DOI: https://doi.org/eid2506.180339

Assessment of Economic Burden of Concurrent Measles and Rubella Outbreaks, Romania, 2011–2012

Appendix

Appendix Table. Diagnostic codes and principal diagnosis used for health insurance reimbursement claim data collection, measles and rubella outbreaks, Romania, 2011–2012

Diagnostic code*	Principal diagnosis
B05.0	Measles complicated by encephalitis
B05.1	Measles complicated by meningitis
B05.2	Measles complicated by pneumonia
B05.3	Measles complicated by otitis media
B05.4	Measles complicated by intestinal complications
B05.8	Measles with other complications
B05.9	Measles without complications
B06.0	Rubella with neurologic complications
B06.8	Rubella with other complications
B06.9	Rubella, no complications
M01.40	Rubella arthritis, multiple localizations
M01.41	Rubella arthritis, shoulder region
M01.42	Rubella arthritis, upper arm
M01.43	Rubella arthritis, forearm
M01.44	Rubella arthritis, hand
M01.45	Rubella arthritis, pelvic region and thigh
M01.46	Rubella arthritis, lower leg
M01.47	Rubella arthritis, ankle and leg
M01.48	Rubella arthritis, another location
M01.49	Rubella arthritis, location unspecified
P35.0	Congenital rubella syndrome
Z24.4	Needed immunization against measles not associated with other diseases
Z27.4	Needed immunization against measles, mumps, rubella

*International Classification of Diseases, 10th revision.

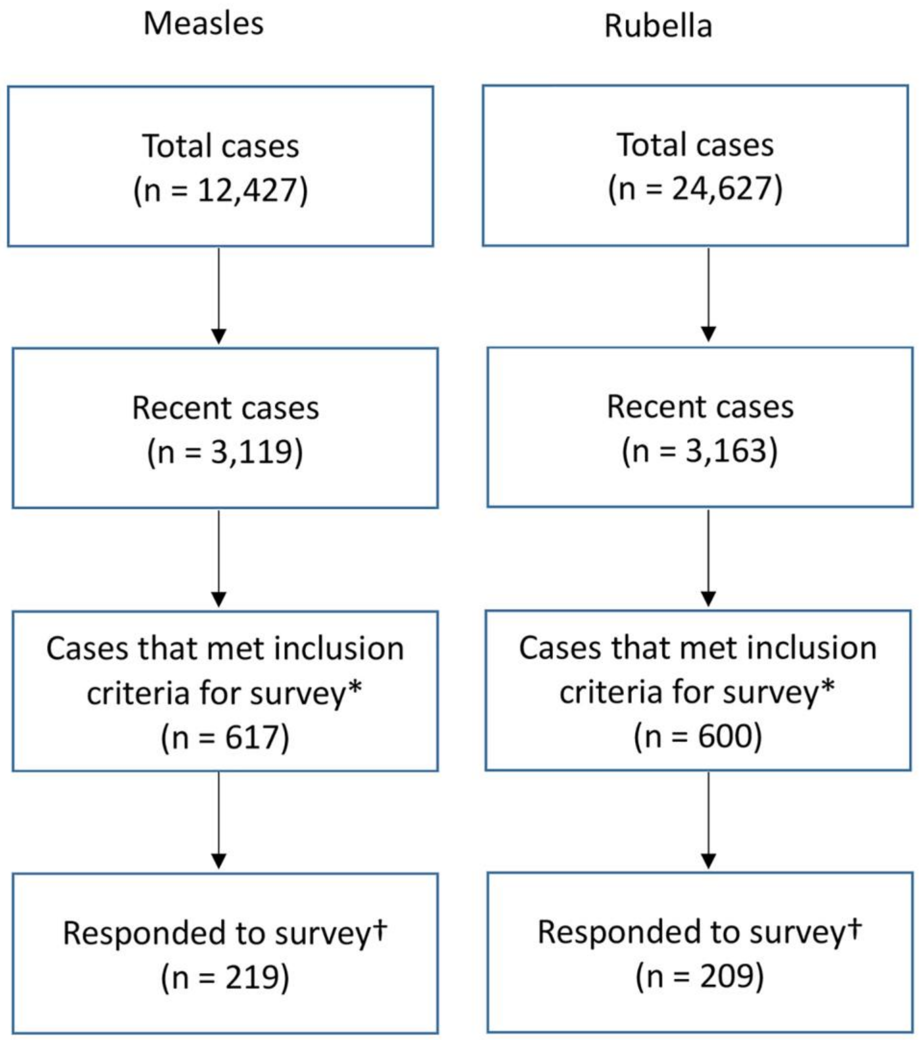

Appendix Figure. Household survey sample selection results, measles and rubella outbreaks, Romania, 2011–2012. *Inclusion criteria for households were 1) reported measles and rubella cases within the previous 18 months of the interview (to minimize recall bias) and 2) availability of critical information for the survey, including a case report, age of the case-patient (specifically, <18 years or ≥18 years), place of residence (urban/rural), and sex. (In Romania, the official age for employment is 18 years.) We intended to interview a minimum of 30 case-patients from each of the following strata for each disease: urban, age <18 years; urban, age ≥18 years; rural, age <18 years; and rural, age ≥18 years. †For case-patients who were unavailable or did not have time to be interviewed on the initial call, a second attempt was made to obtain information the next day or when the respondent indicated he or she would be available for the interview. As a quality control and validity check, 10% of phone questionnaires were randomly sampled, and surveyors conducted in-person interviews to compare data obtained between the 2 methods.
